# Biomedical Ontologies to Guide AI Development in Radiology

**DOI:** 10.1007/s10278-021-00527-1

**Published:** 2021-11-01

**Authors:** Ross W. Filice, Charles E. Kahn

**Affiliations:** 1 Department of Radiology, MedStar Georgetown University Hospital, Washington, DC USA; 2 Department of Radiology and Institute for Biomedical Informatics, University of Pennsylvania, 3400 Spruce Street, Philadelphia, PA 19104 USA

**Keywords:** Artificial intelligence, Ontology, Knowledge representation, Terminology, Controlled vocabulary

## Abstract

The advent of deep learning has engendered renewed and rapidly growing interest in artificial intelligence (AI) in radiology to analyze images, manipulate textual reports, and plan interventions. Applications of deep learning and other AI approaches must be guided by sound medical knowledge to assure that they are developed successfully and that they address important problems in biomedical research or patient care. To date, AI has been applied to a limited number of real-world radiology applications. As AI systems become more pervasive and are applied more broadly, they will benefit from medical knowledge on a larger scale, such as that available through computer-based approaches. A key approach to represent computer-based knowledge in a particular domain is an ontology. As defined in informatics, an ontology defines a domain’s terms through their relationships with other terms in the ontology. Those relationships, then, define the terms’ semantics, or “meaning.” Biomedical ontologies commonly define the relationships between terms and more general terms, and can express causal, part-whole, and anatomic relationships. Ontologies express knowledge in a form that is both human-readable and machine-computable. Some ontologies, such as RSNA’s RadLex radiology lexicon, have been applied to applications in clinical practice and research, and may be familiar to many radiologists. This article describes how ontologies can support research and guide emerging applications of AI in radiology, including natural language processing, image–based machine learning, radiomics, and planning.

## Introduction

The successful development and application of artificial intelligence (AI) in radiology must be guided by medical knowledge. Such knowledge can help AI developers select important clinical problems to solve, identify the limitations of AI solutions, and establish appropriate metrics by which to judge the performance of the solutions. Standard vocabularies, coding systems, and computer-based representations of medical knowledge can promote interoperability and enable sophisticated information systems [1]. To date, many of the applications of AI in radiology have been “artisanal”: they have focused on relatively narrow problems, such as detection of wrist fractures [2] or single neurological abnormalities [3]. As AI becomes more pervasive and more general—including applications in natural language processing (NLP), image–based machine and deep learning, radiomics, and treatment planning—there is a growing need to incorporate knowledge on a larger scale, such as that available through computer-based approaches. This article describes how biomedical ontologies—computer-based representations of knowledge—can help guide emerging applications of AI in radiology.

## Ontology

Ontology is the discipline in philosophy that studies the nature of being; it aims to understand how things in the world are divided into categories and how these categories are related to one another. In its modern meaning related to computing, an ontology describes a structured representation of the knowledge within a certain domain. An ontology classifies the entities within a domain; each entity is said to make up a term, or “class,” of the ontology [4]. An ontology defines a domain’s terms through their relationships with other terms in the ontology; those relationships, then, define the terms’ semantics, or “meaning.” Ontologies express knowledge in a form that is both human-readable and machine-computable [5]. Thus, an ontology can allow both humans and computers to describe and reason about the concepts in a domain. Ontologies help promote clarity and can enable disparate medical systems in radiology to work together [1].

Ontologies are based on controlled vocabularies, several of which will be familiar to radiologists [1]. Current Procedural Terminology (CPT) provides a standardized nomenclature and set of codes for imaging procedures. The International Classification of Diseases, 10th Edition, Clinical Modification (ICD-10-CM) standardizes the names of diseases and medical conditions. Unlike individual terminologies and coding systems, however, ontologies include the semantic relationships between their terms.

We introduce several key ontologies that relate to the development of AI in radiology (Table 1). The ontologies range in size from hundreds of entities to more than 350,000 terms. They range in scope from very narrow to very broad. More than 800 biomedical ontologies are available through the National Center for Biomedical Ontology (NCBO) BioPortal site (9, 10).

**Table 1:** Ontologies and vocabularies relevant to AI applications in diagnostic radiology

Name (abbreviation)	Description	Number of entities	Web site	BioPortal ID*	Reference
Disease Ontology (DO)	A hierarchical vocabulary to classify human disease	12,694	disease-ontology.org	DOID	[14]
Foundational Model of Anatomy (FMA)	A detailed ontology of human anatomy, part of which has been incorporated into RadLex	104,721	si.washington.edu/projects/fma	FMA	[49]
Human Phenotype Ontology (HPO)	A structured and controlled vocabulary of the phenotypic features encountered in human hereditary and other diseases	18,675	human-phenotype-ontology.org	HP	[15]
International Classification of Diseases, 10th Edition, Clinical Modification (ICD-10-CM)^a^	A terminology to classify diagnoses and the reason for visits in all American healthcare settings (2)	95,209	cdc.gov/nchs/icd	ICD10CM	[50]
Logical Observation Identifier Names and Codes (LOINC)^a^	A terminology standard for health measurements, observations, and documents (2)	247,754	loinc.org	LOINC	[51]
Orphanet Rare Disease Ontology (ORDO)	A structured vocabulary of more than 7000 rare diseases with relationships between diseases, genes, and other relevant features	14,501	www.orpha.net	ORDO	[52]
Phenotypic Quality Ontology, formerly Phenotype and Trait Ontology (PATO)	An ontology of phenotypic qualities (properties, attributes, or characteristics)	2805	obofoundry.org/ontology/pato	PATO	[53]
Radiology Gamuts Ontology (RGO)	A set of disorders, interventions, and imaging findings with their causal relations	16,912	gamuts.net	GAMUTS	[13]
Radiology Lexicon (RadLex)	A structured lexicon of radiology terms, including pertinent anatomy, diseases, and imaging findings	46,636	radlex.org	RADLEX	[8]
Radiomics Ontology (RO)	An ontology of radiomics features, segmentation algorithms, and imaging filters	458		RO	[41]
Radiation Oncology Ontology (ROO)	An ontology used to query information from various data structures	1307		ROO	[43]
Radiation Oncology Structures Ontology (ROS)	Anatomic and treatment planning classes that describe commonly contoured structures for treatment planning	417	github.com/jebibault/Radiation-Oncology-Structures-Ontology	ROS	[44]
Systematized Nomenclature of Medicine Clinical Terms (SNOMED CT)	A comprehensive, multilingual clinical healthcare terminology that enables consistent representation of clinical content in electronic health records	357,533	snomed.org	SNOMEDCT	[54]
Units of Measurement Ontology (UO)	Metrical units for use in conjunction with PATO	428	obofoundry.org/ontology/uo	UO	[55]

^*^The “BioPortal ID” references the ontology on the NCBO BioPortal site; for example, the URL for the Disease Ontology (“DOID”) on BioPortal is https://bioportal.bioontology.org/ontologies/DOID

^a^ICD and LOINC are not ontologies. They are controlled vocabularies, and lack relationships between terms

### SNOMED CT

The Systematized Nomenclature of Medicine Clinical Terms (SNOMED CT) is the largest multilingual health terminology. It enables the electronic interchange of health information by assuring consistent representation of clinical content in electronic health records. SNOMED CT is the accepted US standard for health language, and is freely available in the USA through the National Institutes of Health’s National Library of Medicine. SNOMED CT is mapped to other international standards to facilitate semantic interoperability, and it is in use in more than 80 countries.

**Fig. 1:**
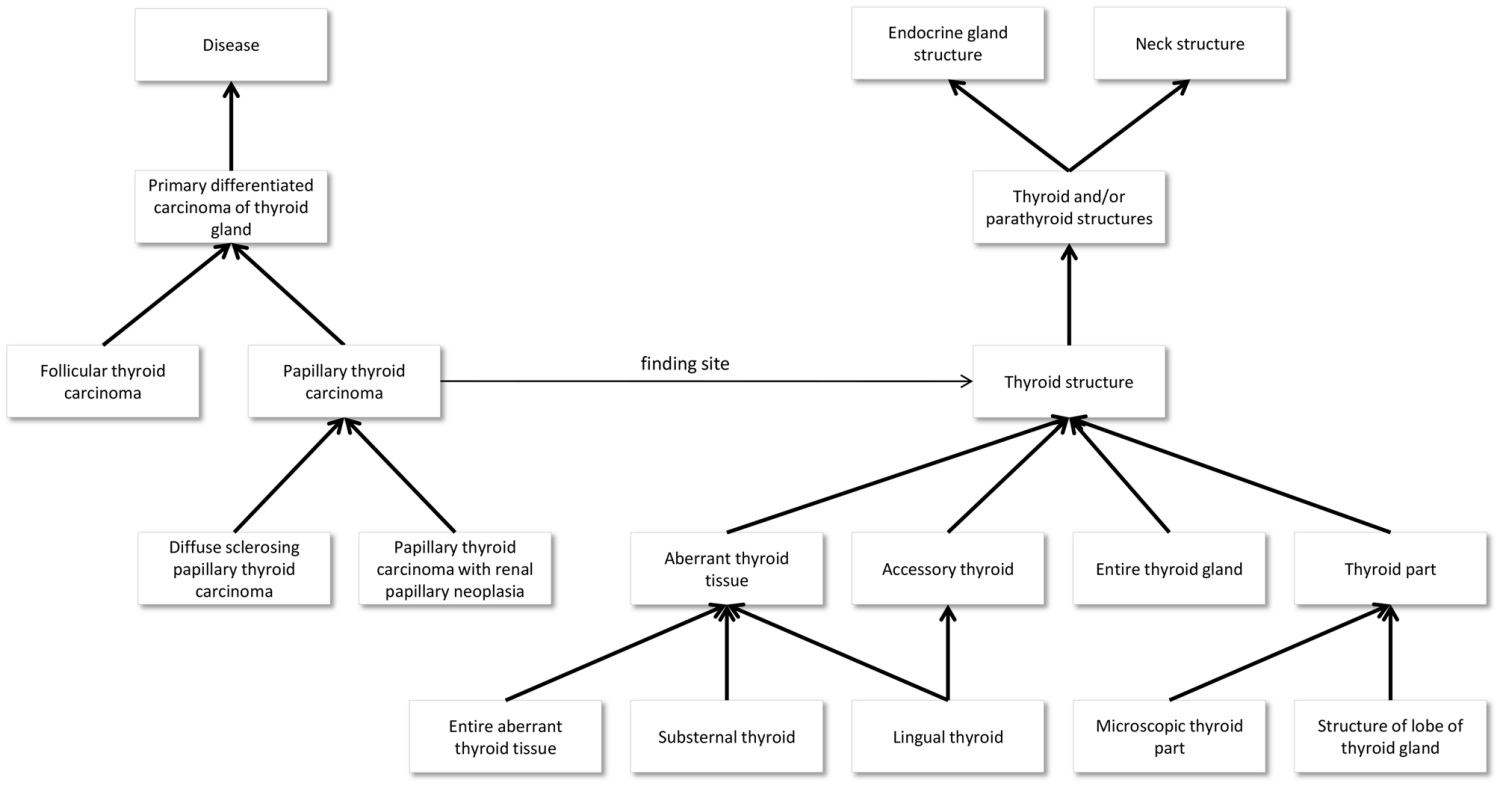
Parts of the SNOMED CT ontology are shown as a directed acyclic graph. The nodes of the graph represent an ontology’s concepts, such as *papillary thyroid cancer*. The *is-a* relationships that relate a more specific concept to a more general one are shown as heavy arrows. The graph also shows the *finding site* attribute, which links a disease or condition to an anatomic structure. Some of the concepts related to *thyroid structure* are presented

Every SNOMED CT concept has a Fully Specified Name (FSN), a unique, unambiguous description of a concept’s meaning. The FSN is particularly useful when different concepts are referred to by the same commonly used word or phrase. A synonym represents a term that can be used to display or select a concept. A concept may have several synonyms, which allows one to use the term one prefers for a specific clinical meaning. For example, concept 22298006 has the fully specified name *myocardial infarction* (*disorder*), and synonyms such as *myocardial infarction*, *heart attack*, and *MI*. Top-level classes in SNOMED CT with the greatest number of subclasses are *body structure*, *clinical finding*, *organism*, *pharmaceutical/biologic product*, *procedure*, and *substance*. A part of the SNOMED CT hierarchy is shown in Fig. 1.

A relationship represents an association between two concepts. Relationships define the meaning of a concept in a way that can be processed by a computer. The relationship type (or *attribute*) specifies the meaning of the association between the source and destination concepts. There are different types of relationships available within SNOMED CT. For example, the *is-a* attribute links *diabetes mellitus type 2* to *diabetes mellitus*: it expresses that the former is a subtype of the latter. Here are the defined relationships for *Pancreas divisum*:

*Pancreas divisum*

Synonyms   *Pancreatic divisum*

Is-a   *Congenital malformation of pancreas*

Has-associated-morphology   *Developmental failure of fusion*

Has-finding-site   *Pancreatic structure*

In addition to primitive concepts, some SNOMED CT terms are defined logically in relation to other terms. For example, *viral upper respiratory tract infection* depicts a fully described concept, which is represented in description logic as a logical conjunction:

*Viral upper respiratory tract infection*

equivalentTo

 *Upper respiratory infection* 

 and  *Viral respiratory infection* 

 and  Causative-agent some Virus 

 and  Finding-site some *Upper respiratory tract structure*

 and  Pathological-process some *Infectious process * 

#### RadLex

**Fig. 2:**
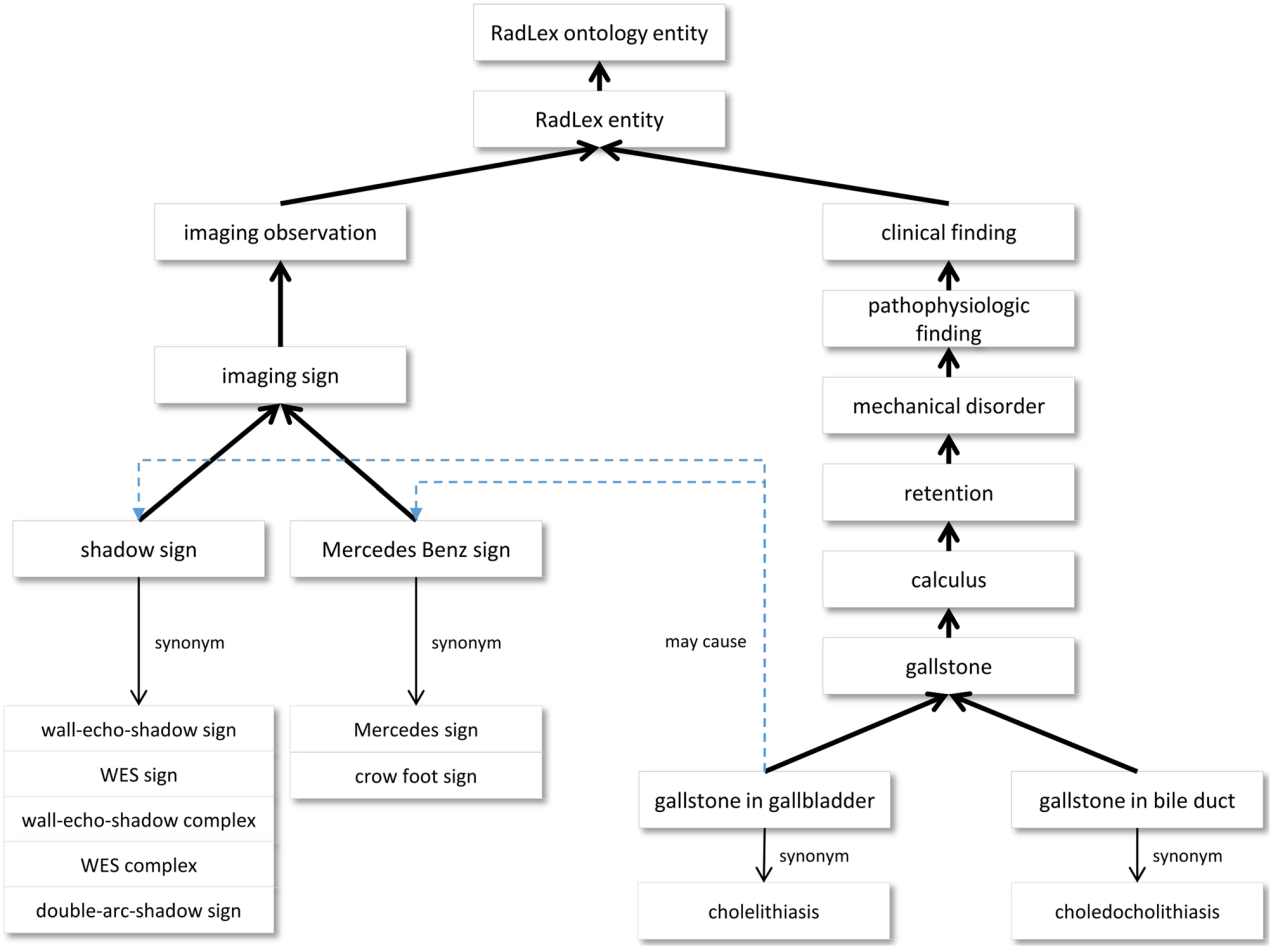
Example of RadLex concepts. One can view the hierarchy of concepts related to *gallstone in gallbladder* and its associated imaging signs

The RadLex radiology lexicon has been developed to create a uniform, consistent language for radiology to improve communication of results and to better integrate clinical practice with education and the scientific literature. RadLex was created, in part, to address the lack of radiology-specific terms in a general medical vocabulary such as SNOMED CT [6]. As an ontology of radiology, RadLex terms describe relevant anatomy, diseases, imaging findings, procedures, and other concepts of use in radiology practice [7, 8]. RadLex has 15 top-level concepts, including *anatomical entity*, *clinical finding*, and *imaging observation* (Table 2). RadLex incorporates concepts and relations from the Foundational Model of Anatomy (FMA), a detailed ontology of human anatomy [9]. In addition to the typical class-superclass relationships (*is_a* and its inverse *has_subtype*) and part-whole relationships (*part_of* and its inverse *has_part*), RadLex includes a rich set of relationships, largely derived from FMA, that express relationships such as the anatomical site of a finding or disease; muscle origin, insertion, and innervation; and vascular anatomy. RadLex incorporates frequently used synonyms, and has been translated into German (Fig. 2).

**Table 2:** Top-level concepts of the RadLex ontology. A descendant is any concept directly or indirectly specified as a subclass (or “child”)

Top-level concept	Number of descendants
Anatomical entity	38,165
Clinical finding	2230
Imaging observation	1134
Imaging specialty	86
Non-anatomical substance	392
Object	403
Procedure	610
Procedure step	98
Process	35
Property	1308
RadLex descriptor	1311
RadLex non-anatomical set	7
Report	0
Report component	22
Temporal entity	4

RadLex also provides a consistent nomenclature for radiology procedures to use in exam ordering, scheduling, billing, and image interpretation. Standardized procedure codes promote interoperability, facilitate the identification of relevant prior imaging studies, and enable data to be collected in national registries, such as the American College of Radiology’s Dose Index Registry. RadLex procedure names have been integrated with the Logical Observation Identifiers Names and Codes (LOINC) standard, a widely used vocabulary for laboratory procedures and results (https://loinc.org), to offer the LOINC-RSNA Radiology Playbook as a uniform scheme for imaging procedure names [10, 11].

#### Radiology Gamuts Ontology

**Fig. 3:**
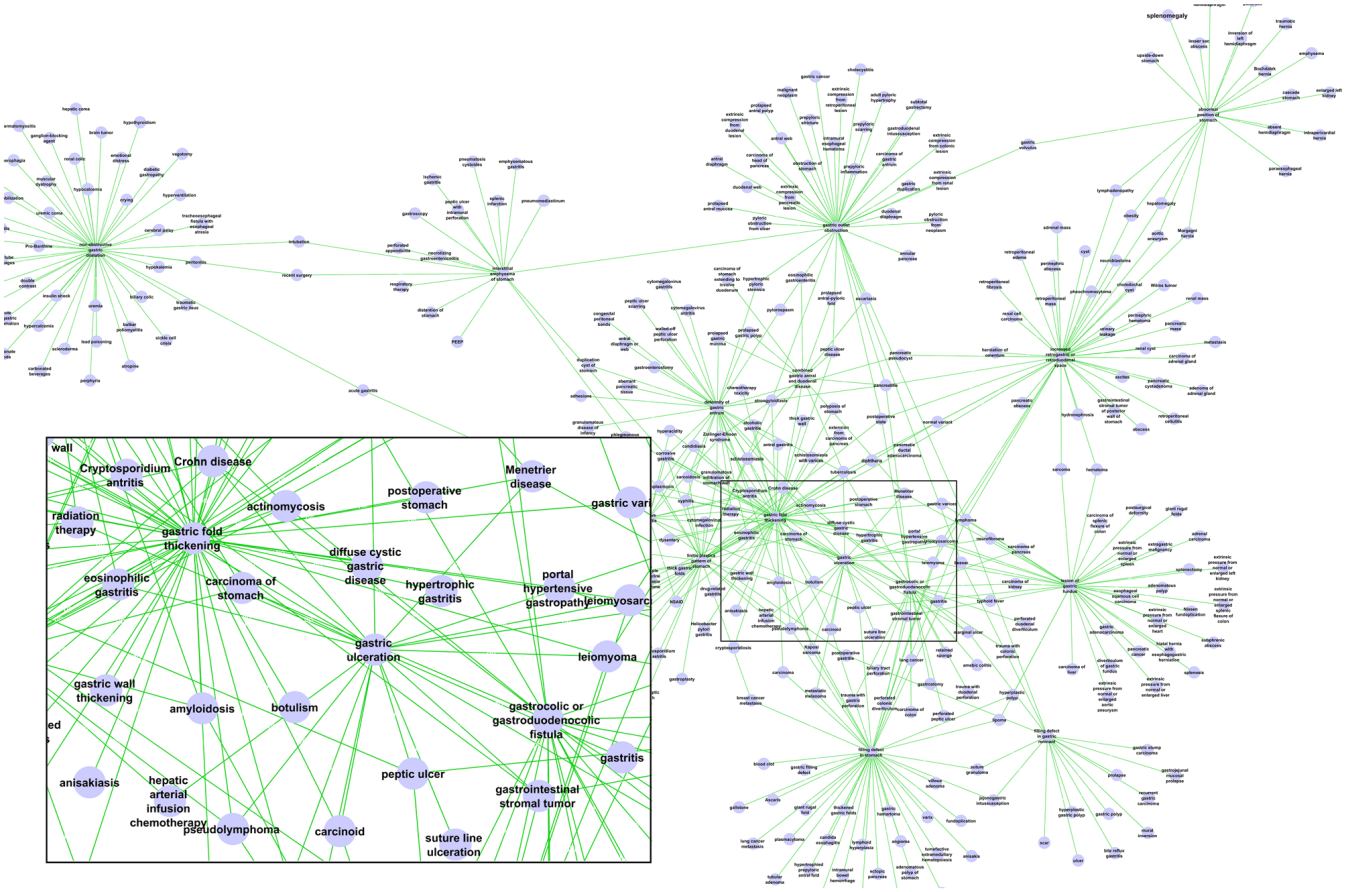
A portion of the network of Radiology Gamuts Ontology (RGO) terms and their causal relationships displayed as a graph, limited to a subset of conditions related to the stomach. RGO concepts, such as *gastric fold thickening*, are the nodes, shown as solid blue circles. The green arcs between nodes represent causal relationships. The inset at lower left provides a magnified view of a demarcated portion of the graph

Differential diagnosis has formed the core of traditional knowledge in radiology, and various reference texts have provided knowledge of differential diagnosis for clinical radiology practice, such as *Reeder and Felson’s Gamuts in Radiology: Comprehensive Lists of Roentgen Differential Diagnosis* [12]. Knowledge of radiological differential diagnosis has been incorporated into the Radiology Gamuts Ontology (RGO). RGO comprises 16,912 concepts that specify disorders (e.g., *Apert syndrome*), interventions (e.g., *Whipple procedure*), and imaging manifestations (e.g., *gastric fold thickening*) [13]. In addition to the conventional hierarchical (“is a”) relation between more specific and more general concepts, RGO defines the “may cause” relation (and its inverse, “may be caused by”) that encodes links between conditions and their imaging manifestations. For example, RGO asserts that *gastric fold thickening* may be caused by *gastric varices*, *Ménétrier disease*, and 46 other conditions. RGO terms—together with their 1782 hierarchical (“is a”) and 55,564 causal relationships—form a large, interconnected network of knowledge for radiological diagnosis (Fig. 3). In addition to publication on the NCBO BioPortal site and a custom web site, an application programming interface (API) makes the ontology’s knowledge available in machine-readable form. Grouping of RGO terms by organ system and imaging modality reveals the breadth of content in each subdomain (Table 3).

**Table 3:** Number of imaging findings and diseases defined in the Radiology Gamuts Ontology by organ system and imaging modality. Entities may be listed in one or more categories

Category	Number of imaging findings	Number of diseases
Breast imaging	2	60
Cardiac radiology	106	1178
Chest radiology	286	2958
Computed tomography	1232	9081
Diagnostic radiology	1432	9266
Gastrointestinal radiology	363	2869
Genitourinary radiology	236	1901
Head and neck radiology	272	2627
Musculoskeletal radiology	719	4069
Magnetic resonance imaging	1042	6947
Neuroradiology	462	2767
Obstetric/gynecologic radiology	72	653
Oncologic imaging	65	736
Pediatric radiology	198	2449
Ultrasound	499	4605
Vascular imaging	172	1809

#### Disease Ontology

The Disease Ontology (DO) is an extensive, hierarchically organized vocabulary of 12,694 human diseases that provides a framework to identify relationships between diseases and phenotypes, genotypes, and various other disease attributes [14]. DO provides semantically consistent annotations that allow one to compare diagnostic evaluations, treatments, and patient-care data over time and between studies. DO incorporates concepts and extensive cross-mapping from standard clinical and medical terminologies, such as Medical Subject Headings (MeSH), International Classification of Diseases (ICD), Online Mendelian Inheritance in Man (OMIM), and the National Cancer Institute Thesaurus. The ontology provides a resource to connect genetic and phenotypic information related to human disease.

#### Human Phenotype Ontology

The Human Phenotype Ontology (HPO) describes phenotypic features of hereditary, congenital, and acquired diseases using a structured and controlled set of terms [15]. Although focused initially on monogenic diseases—about 50,000 annotations connect HPO terms to 4779 diseases in the OMIM database of genetic disorders—HPO now includes features of more than 3400 common non-Mendelian disorders. HPO terms can have more than one parent in the phenotypic hierarchy: for example, *podagra* (gout of the big toe) has parent terms *gout* and *abnormality of the foot*. HPO has been linked to OMIM and to the Orphanet Rare Disease Ontology (ORDO) to increase the interoperability of phenotypic knowledge in rare diseases. One can match clinical information to phenotypes at varying levels of specificity in the ontology’s hierarchy to formulate differential diagnoses; for example, a clinical record describing a “short 2nd toe” would be linked to the HPO term *short 2nd toe* and its more general terms, *short toe*, *short digit*, and *abnormal digit morphology.*

#### Integration of Ontologies

**Fig. 4:**
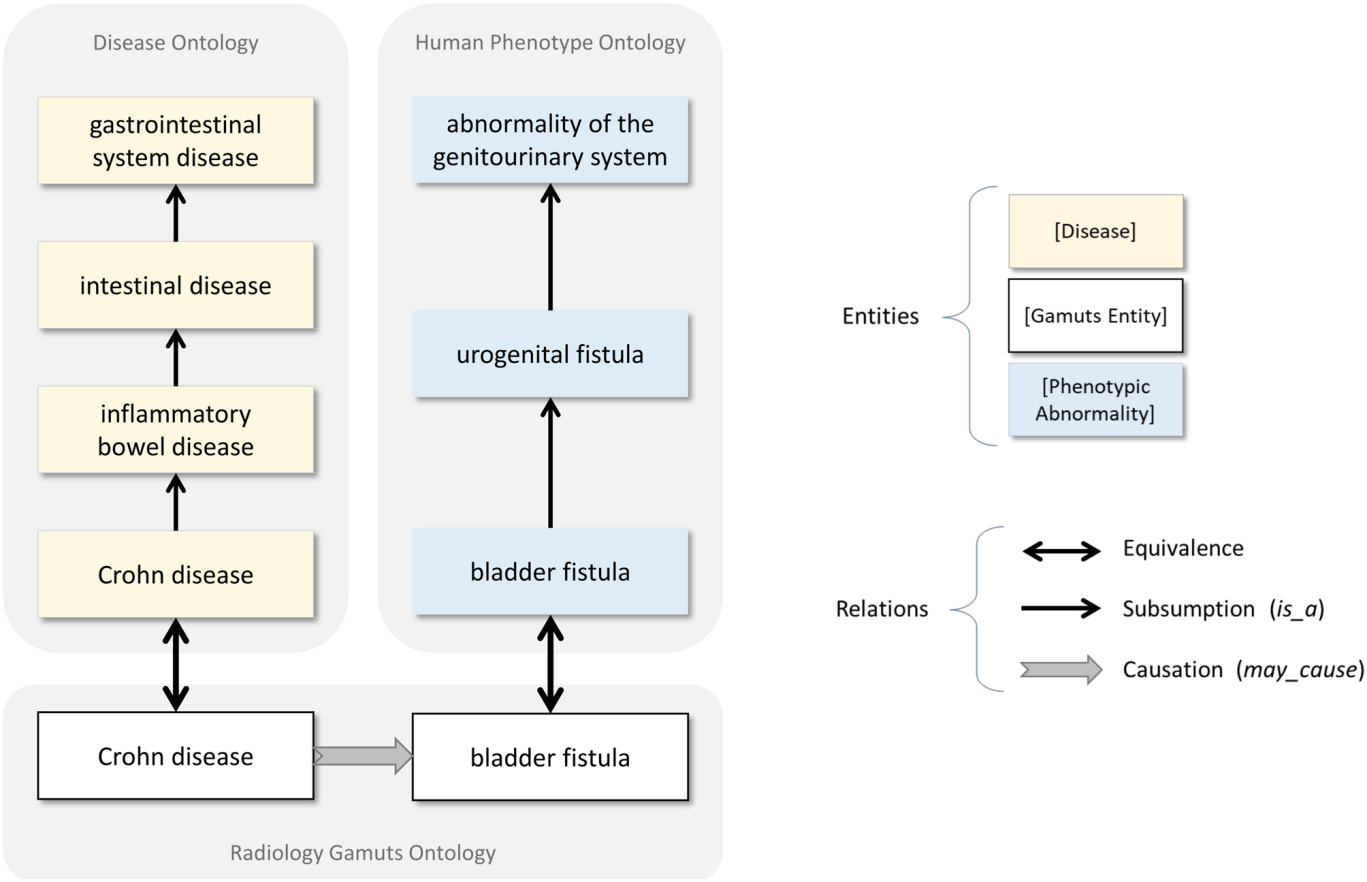
Radiology Gamuts Ontology’s causal knowledge and mappings to the Disease Ontology and Human Phenotype Ontology allow one to answer questions such as, “Which gastrointestinal disease(s) may cause an abnormality of the genitournary system?” The example presented shows the causal relationship from *Crohn disease* to *bladder fistula*, with corresponding hierarchical relationships of diseases and phenotypic abnormalities

Individual ontologies can serve as building blocks of broader, more general knowledge resources. By integrating related ontologies, knowledge can be shared and reused across domains. The US National Library of Medicine’s Unified Medical Language System (UMLS) Metathesaurus seeks to provide semantic integration of concepts across ontologies and vocabularies; a single concept unique identifier (CUI) in the UMLS Metathesaurus may refer to concepts in several component vocabularies. As described above, the Foundational Model of Anatomy (FMA) forms much of the basis of the anatomical terms in RadLex. Knowledge of radiological differential diagnosis in RGO has been integrated with SNOMED CT, RadLex, DO, HPO, and the ORDO [16–18]. This integration allows one to pose new, abstract questions that relate diseases and their imaging phenotypes such as, “Which gastrointestinal system diseases may cause an abnormality of the genitourinary system?” (Fig. 4).

#### Limitations

Although ontologies provide a powerful framework to organize information, they have several limitations both in their design and application. With more than 800 ontologies available through NCBO BioPortal (https://bioportal.bioontology.org), it can be challenging to discover new or relevant ontologies. Mappings between these ontologies are sparse and few are included in the UMLS Metathesaurus. SNOMED CT, LOINC, and ICD-10-CM are part of the Metathesaurus, but, notably, RadLex is not. Licensing of ontologies can pose another barrier: the US federal government licenses SNOMED CT for use in the USA, but the ontology is not openly available worldwide. Uneven or incomplete development of ontologies can limit their use: many ontologies, including RadLex, are built either through volunteer effort or are part of a larger initiative. Only a minority of RadLex terms include a definition; new concepts that enter the vocabulary of radiology may not yet be incorporated, and there are ongoing questions of the scope and purpose of any ontology. As ontologies grow larger, it is increasingly challenging to assure that they are maintained appropriately and that the ontology’s axioms are logically consistent.

Few ontologies other than SNOMED CT and LOINC have been incorporated broadly into commercial products, and solutions adopted in industry often resort to simpler, non-standards-based textual information. Towbin et al. have highlighted the widespread and substantial limitations of traditional free-text body part labeling in DICOM and the importance of an ontology for body part labeling to enable enterprise imaging [19]. Smith et al. review many of the challenges of ontologies for biomedical imaging, and propose a coordinated suite of ontologies to address the needs of biomedical imaging [20]. There may be lack of incentive in the commercial space as organizing or labeling data ontologically requires more work and maintenance; it is unclear what might motivate such adoption.

## Applications

### Natural Language Processing

Natural language processing (NLP) has been applied widely in radiology, and can perform more efficiently than human experts with near-human accuracy [21–23]. Many clinical and research applications rely on automatic recognition of medical concepts in unstructured text, and the accuracy of recognition can affect the analysis of electronic health records. The mining of medical concepts is complicated by the broad use of synonyms and nonstandard terms in medical documents. Ontologies have been shown to improve performance of NLP applications [24–26].

Ontologies can support radiology NLP applications by providing a comprehensive vocabulary of a domain, along with the terms’ synonyms, abbreviations, and relationships. Ontology’s synonyms, abbreviations, and related terms can help identify diagnoses and other entities in radiology reports. For example, RGO incorporates 2957 synonyms (e.g., *hepatoma* and *HCC* for *hepatocellular carcinoma*) that can improve recall of an NLP search routine and can also help coalesce the findings into a single unique entity. Links between ontologies can offer additional synonyms; for example, the RGO term *LEOPARD syndrome* has DO synonyms: *Capute-Rimoin-Konigsmark-Esterly-Richardson syndrome*, *generalized lentiginosis*, *Gorlin syndrome II*, *lentiginosis profusa syndrome*, *Moynahan syndrome*, *multiple lentigines syndrome*, *Noonan syndrome with multiple lentigines*, and *progressive cardiomyopathic lentiginosis*. Hierarchically organized information allows one to search intelligently for classes of findings; for example, a search for *phakomatosis* encompasses more specific terms such as *neurofibromatosis*, *NF1*, and *tuberous sclerosis*.

Text de-identification tools often use algorithms that attempt to redact people’s names by identifying words that are capitalized. Radiology reports, however, commonly contain eponyms for devices, findings, and diseases that include capitalized proper nouns (e.g., *Foley catheter*, *Kerley B lines*, *Wilms tumor*). Thus, de-identification tools may require domain-specific vocabulary [27, 28] so that these terms are not inappropriately redacted from the record or replaced by arbitrary names as in de-identification techniques such as “hiding in plain sight” [29]. More than 800 of these imaging findings (such as *Rigler sign*) have been incorporated into RadLex [30]. Connections between ontologies can identify additional similarly capitalized proper noun synonyms as alluded to in more detail above. Although modern neural network–based approaches have reduced the need for feature engineering, exposure to controlled vocabularies can still supplement these techniques and limit the need for extremely large sets of training data [31].

In addition to search-and-replace tasks and named-entity recognition, ontologies can supplement deep neural network–based techniques by creating word embeddings based on ontology terms. An embedding technique converts words into vectors, or numerical representations, based on neighboring and co-occurring words in a text corpus. SNOMED2Vec, a word embedding model that uses SNOMED CT to augment machine-defined relationships, can outperform models that are not based on ontologies because it strengthens complex medical relationships such as “breast + adenocarcinoma → invasive ductal carcinoma” [32].

The relationships in an ontology enable more robust medical reasoning. The multiple hierarchical levels of terms in an ontology enable connections that go beyond word-list associations (e.g., for a synonym or proper noun detection) to make an end model more medically relevant. Researchers have explored semiautomated approaches to learn ontologies from text [33]. NLP performance was improved on a gene/protein synonym detection task by adding formal ontological knowledge without modifying the word embeddings; the ontology provided context that effectively induced term variability while reducing ambiguity [34]. Biomedical ontologies evolve continually to become more robust and to accommodate new imaging techniques and findings. Ontologies can augment NLP, and NLP can be used to analyze free-text corpora to find gaps and to suggest terms to add to existing ontologies [35].

## Image–Based Machine Learning

Image–based machine learning can be applied to a virtually infinite space of potential applications across diseases, findings, and imaging modalities. Early successful image–based machine learning applications have focused narrowly on particular findings or diseases; a key challenge is to identify the applications that will have the greatest impact on radiologists’ performance and patient care. Ontologies can help guide investigators towards fruitful avenues for research. For example, musculoskeletal radiology alone entails 635 differential-diagnosis listings in RGO, such as *abnormal odontoid process* and *premature craniosynostosis*, with their interlinked imaging findings and diseases. The differential-diagnosis listing for *abnormal odontoid process* links to 37 possible causes, including *ankylosing spondylitis* and *systemic lupus erythematosus*. Similarly, in thoracic imaging, RGO includes 286 imaging findings caused by 2958 diseases (Table 3). Understanding these linkages can guide researchers to focus on findings that are intertwined with diseases to increase the chance of substantive clinical impact.

These hierarchical relationships and mappings can then help translate findings that are detected using image-based techniques to diseases or other entities [36] which may strengthen an image–based machine learning model and help with one key area of machine learning, explainable or interpretable AI [37, 38]. Image-based models may use saliency maps (“heat maps”) or activation maps to explain which image features contributed most strongly to certain findings in an exam. Ontologies and their relationships can then help synthesize these findings to propose causal relationships or subsequent diseases and outcomes and can show the mappings that led to these conclusions. For example, an image-based AI model may detect cirrhosis; ontological knowledge can suggest what might have caused the finding (alcohol abuse, viral hepatitis, etc.) and possible consequences (portal hypertension, hepatocellular carcinoma, etc.).

One of the most challenging and time-consuming parts of developing image–based machine learning tools is labeling data. Early approaches typically required cumbersome manual labeling by domain experts, but automated or semiautomated techniques that generate large corpora of slightly imperfect training data can approach and nearly match the performance from carefully manually curated labels, although weakly labeled datasets generally require significantly more labels to approach the same level of performance [39], Ontologies can support the development of datasets to train or test image-based AI models by identifying mentions of conditions or imaging findings in radiology reports.

High-quality NLP models can facilitate important image–based machine learning applications and their evaluation. Marrying an image-based model that detects certain findings or entities with an NLP model that can accurately identify those findings in the report could be used to automate peer review at a scale far greater than what is feasible with current manual methods. Machine learning tools used by a practice should be continually monitored for performance and for safety events; parsing report data for the findings suggested by the image-based model can help quantify the performance and potential errors of both AI systems and humans.

## Radiomics

Radiomics can link the characteristics of radiological images with a patient’s genotype, gene expression profile, and prognosis [40, 41]. The use of ontologies can help identify the scope of diseases or imaging findings related to a particular radiology exam and guide the integration of radiomics features with specific diseases. The Image Biomarker Standardization Initiative (IBSI) has defined uniform, reproducible approaches to compute 174 quantitative features from medical images [42]. The IBSI radiomics features have been integrated with segmentation algorithms and imaging filters into the Radiomics Ontology, which is published through NCBO BioPortal [43]. An ontology-guided radiomics analysis workflow has been developed to capture features from imaging data to facilitate research and clinical translation of radiomics [44]. As radiomics methods become more commonplace and sophisticated, one can imagine that particular computational features will become associated with various conditions, and such information could be incorporated into a diagnostic ontology.

## Radiation Therapy Planning

Ontologies can aid in planning, which is the task of identifying optimal sequences of actions to achieve a specified goal. An example of planning is found in mapping applications that can identify the fastest route to a given destination. In diagnostic radiology, a planning task might be to identify the most cost-effective imaging procedures to answer a clinical question. A common planning task in radiation oncology is to select optimal radiation ports to treat target lesions while sparing nontarget organs.

Two ontologies have been developed in radiation oncology. The Radiation Oncology Ontology (ROO) represents clinical data in the radiation oncology domain; it incorporates 1183 classes and 211 properties between classes [45]. The authors used the ontology to query information from various data structures without a priori knowledge of the schemas of the underlying relational databases. The Radiation Oncology Structures (ROS) ontology comprises 417 anatomic and treatment planning classes that describe commonly contoured structures for radiation treatment planning [46]. The ontology was derived from more than 22,000 structure labels used in one radiation oncology department; international guidelines were used for lymph node delineation. ROS was created to standardize radiation oncology data for integration of into clinical data warehouses for multicenter studies.

## Conclusion

Many of the recent advances of AI in medical imaging have focused on the use of machine learning, and in particular, the technique of deep neural networks or deep learning. These systems can learn to recognize features and patterns in radiological images and textual reports to aid in detection and diagnosis. Although researchers have achieved a number of notable early successes, the AI models typically address a small number of conditions. The large number of radiologically relevant conditions and imaging findings for each combination of imaging modality and body part (chest CT, for example) suggests that there is an opportunity to explore the development of “industrial-scale” approaches in which computer-based knowledge serves to relate imaging findings to corresponding diseases. The knowledge represented in ontologies can be used to help provide the “semantics” or meaning that may allow deep learning models to explain their reasoning. For analysis of radiology reports and other textual information in the electronic health record, ontologies provide a rich source of synonyms, and allow one to capture relevant features at various levels of generalization. Ontologies promote integration and interoperability among clinical, imaging, and “omics” data, and support deep learning algorithms, bioinformatics pipelines, big data analyses, and quality assurance and safety initiatives [47, 48]. Knowledge resources such as biomedical ontologies are poised to help guide large-scale foundational and translational research endeavors in radiology AI [49, 50].

## Abbreviations

AI: artificial intelligence
CUI: concept unique identifier
DO: Disease Ontology
FMA: Foundational Model of Anatomy
HPO: Human Phenotype Ontology
IBSI: ICD-10-CM, International Classification of Diseases, 10th Edition, Clinical Modification
LOINC: Logical Observation Identifier Names and Codes
NLP: natural language processing
ORDO: Orphanet Rare Disease Ontology
RGO: Radiology Gamuts Ontology
RO: Radiomics Ontology
ROO: Radiation Oncology Ontology
ROS: Radiation Oncology Structures ontology
SNOMED CT: Systematized Nomenclature of Medicine Clinical Terms
UMLS: Unified Medical Language System
