# Correction to: Journal of Digital Imaging

**DOI:** 10.1007/s10278-021-00568-6

**Published:** 2022-07-15

**Authors:** Ross W. Filice, Charles E. Kahn

**Affiliations:** 1 Department of Radiology, MedStar Georgetown University Hospital, Washington, DC USA; 2 Department of Radiology and Institute for Biomedical Informatics, University of Pennsylvania, 3400 Spruce Street, Philadelphia, PA 19104 USA

## Correction to: Journal of Digital Imaging https://doi.org/10.1007/s10278-021-00527-1

The article “Biomedical Ontologies to Guide AI Development in Radiology,” written by Ross W. Filice and Charles E. Kahn, was originally published Online First without Open Access. After publication in volume 34, issue 2, page 367-373, the author decided to opt for Open Choice and to make the article an Open Access publication. Therefore, the copyright of the article has been changed to ©The Authors, 2021, and the article is forthwith distributed under the terms of the Creative Commons Attribution.

